# CHARITY: Chagas cardiomyopathy bisoprolol intervention study: a randomized double-blind placebo force-titration controlled study with Bisoprolol in patients with chronic heart failure secondary to Chagas cardiomyopathy [NCT00323973]

**DOI:** 10.1186/1745-6215-7-21

**Published:** 2006-06-09

**Authors:** Franklin R Quiros, Carlos A Morillo, Juan P Casas, Luz A Cubillos, Federico A Silva

**Affiliations:** 1 Research Institute, Fundación Cardiovascular de Colombia (FCV), Floridablanca, Santander, Colombia; 2 Department of Medicine, Cardiology Division, Arrhythmia Service, McMaster University, Hamilton, Ontario, Canada; 3 Epidemiology and Population Health Department, London School of Higiene and Tropical Medicine, University of London, UK

## Abstract

**Background:**

Chagas' disease is the major cause of disability secondary to tropical diseases in young adults from Latin America, and around 20 million people are currently infected by *T. cruzi*. Heart failure due to Chagas cardiomyopathy is the main clinical presenation in Colombia. Heart failure due to Chagas' disease may respond to digoxin, diuretics and vasodilator therapy. Beta-adrenoreceptor antagonism seems to protect against the increased risk of cardiac arrhythmia and sudden death due to chronic sympathetic stimulation. The aim of this study is to evaluate the effects of the selective beta-adrenergic receptor blocker Bisoprolol on cardiovascular mortality, hospital readmission due to progressive heart failure and functional status in patients with heart failure secondary to Chagas' cardiomyopathy.

**Methods/design:**

A cohort of 500 *T. cruzi *seropositive patients (250 per arm) will be selected from several institutions in Colombia. During the pretreatment period an initial evaluation visit will be scheduled in which participants will sign consent forms and baseline measurements and tests will be conducted including blood pressure measurements, twelve-lead ECG and left ventricular ejection fraction assessment by 2D echocardiography. Quality of life questionnaire will be performed two weeks apart during baseline examination using the "Minnesota living with heart failure" questionnaire. A minimum of two 6 minutes corridor walk test once a week over a two-week period will be performed to measure functional class. During the treatment period patients will be randomly assigned to receive Bisoprolol or placebo, initially taking a total daily dose of 2.5 mgrs qd. The dose will be increased every two weeks to 5, 7.5 and 10 mgrs qd (maximum maintenance dose). Follow-up assessment will include clinical check-up, and blood collection for future measurements of inflammatory reactants and markers. Quality of life measurements will be obtained at six months. This study will allow us to explore the effect of beta-blockers in chagas' cardiomyopathy.

## Background

Chagas' disease (CD) is a permanent threat for almost a quarter of the population of Latin America. Although the disease has been described in almost all Central and South America, clinical presentation and epidemiological characteristics are variable among the different endemic zones [1,2]. A wide range of prevalence rates has also been reported suggesting local differences in transmission of the disease as well as differences in vectors and reservoirs [3].

Chagas' cardiomyopathy (CCM) represents a serious public health problem in most Latin American countries, and the most recent statistics provided by the World Health Organization indicate that 100 million persons are exposed to the disease and approximately 20 million are currently infected [4]. Interestingly, in addition to the natural infection foci, an increase in the transmission associated with blood transfusions has also been noticed. These statistics are considered an underestimation of the real rates of infection, most likely due to lack of reports from highly endemic retired rural communities. In countries in which the disease is endemic such as Colombia, Venezuela and Brazil, the overall prevalence of infection averages 10%. However, in highly endemic rural areas rates have ranged from 25% to 75% [5]. Prevalence of infection varies widely even between cities and provinces within the same country because of variations in climate, housing condition, public health measures, and urbanization. The actual prevalence of clinical Chagas' disease and the number of case fatalities are largely unknown, mainly because case reporting is virtually nonexistent in many areas in which CD is highly endemic.

Congestive heart failure (CHF) is a late manifestation of CD that results from structural abnormalities and extensive and irreversible damage to the myocardium. Heart failure in *T. cruzi *infected patients usually occurs after age 40 and follows AV block or ventricular aneurysm. However, when CHF develops in patients less than 30 years old it is frequently associated with a more aggressive myocarditis and an extremely poor prognosis [1]. The mortality attributable to CD is related to the severity of the underlying heart disease. Very high mortality is often found in patients with CHF [2], however, mortality in asymptomatic seropositive patients varies greatly between geographic regions, suggesting that other factors may influence the severity and progression rate of cardiac disease. It is believed that cardiac damage in CD progresses slowly but steadily over decades, from subclinical myocarditis to mild segmental abnormalities with conduction defects, to severe ventricular structural abnormalities, and finally to overt congestive heart failure and sudden cardiac death. Besides the poor prognosis of CHF due to Chagas' disease, it is important to estimate the risk of complications and death in patient infected with *T. cruzi*. Unfortunately, few clinical studies have addressed this issue. Most *T. cruzi *infected patients have mild or no clinical disease, however, the percentage of infected people that will develop detectable cardiac abnormalities is approximately 30 to 40% [3], but only 20% of them will develop symptomatic cardiac involvement [6]. Like CHF from other causes, CHF due to CD responds to digital, diuretics and vasodilators therapy [7]. Additionally, some studies have shown that angiotensin-converting enzyme (ACE) inhibitors improve survival in patients with moderate to severe CHF due to CD [8]. In spite of its benefits on patients with non Chagas' disease CHF, there is considerable uncertainty about the potential role of ACE inhibitors in patients with CHF due to Chagas' disease. *Captopril*, and ACE inhibitors, has been shown to reduce neurohormonal activation and non-lethal arrhymias in a small number of patients with Chagas' heart failure [8,9].

Another intervention currently included in the management of CHF patients is the use of b blockers. Observational studies [10] as well as clinical trials [11,12] have shown that b-blockers reduce morbidity and mortality in CHF patients. The effects of b-blockers on CHF patients are being studied in large scale clinical trials [13-19]. Apparently, the cardiac sympathetic hyperstimulation that initially helps to preserve ventricular function in CHF patients, later on, results in an increased risk of cardiac arrhythmia and sudden death [20] b-adrenoreceptor antagonism seems to protect against the deleterious effects of chronic sympathetic stimulation [21]. Moreover, b-blockers reduce heart rate, improve myocardial energetic balance and lead to a less negative force-frequency relationship. These effects contribute to the benefits of b-blocker therapy in CHF patients.

Clinical studies with Carvedilol, an alpha-1 and non selective b-blocker, further support the beneficial effects of b-adrenoceptor antagonism [18,19,23,24]. Results from the US CARVEDILOL and COPERNICUS trial, suggest that prognosis in CHF may partly depend on left ventricular dysfunction improvement as well as sympathetic activity reduction [18,23]. However, additional data are needed to define more precisely the relationships between heart rate reduction, left ventricular function improvement and survival in CHF patients. In some studies like CIBIS II and MERIT HF, the beneficial effects of the selective b- blockers on morbidity and mortality were observed specially in patients functional class II and III. In COPERNICUS a multicentric placebo controlled clinical trial with *Carvedilol*, these effect was ascertained even in patients functional class IV [23], demonstrating that patients with CHF, independently of their functional class, should receive b-blocker therapy.

Current guidelines for the management of CHF strongly recommend the use of beta-blockade in management of CHF. However, these benefits have not been proven in Chagas' cardiomyopathy [25,26]. Nevertheless, there are reasons to believe that beta-blockade will be beneficial for these patients.

First, patients with CHF due to CD have a raised end-diastolic pressure associated with a low systemic blood pressure, which lead to low transmyocardial pressure gradient and subendocardial ischemia. Therefore, reduction in myocardial oxygen demand due to beta-blockade could be expected to be beneficial in this situation, even in non ischemic CHF patients. Second the reduction in sudden cardiac deaths and serious ventricular arrhythmias suggest that an anti arrhythmic effect is an important component of beta-blockade. This anti-arrhythmic effect is explicable not only on an anti-ischemic basis but also by blockade of sympathetic activity, which is indeed increased in patients with Chagas' cardiomyopathy. Third, prolonged activation of the sympathetic nervous system can accelerate the progression of heart failure, and the rate of progression can be substantially decreased using pharmacological agents that interfere with sympathetic activity in the heart and peripheral blood vessels. There is clear evidence of increased circulating beta-adrenergic antibodies in Chagas' disease providing further theoretical support for the use of beta-adrenergic blockade in this group of patients.

Beta-blockers already proven to be beneficial in CHF patients are metoprolol, bisoprolol and carvedilol, drugs which have been tested in over 10.000 patients in more than 20 placebo-controlled clinical trials enrolling men and women with systolic dysfunction due to ischemia, hypertension and dilated idiopathic cardiomyopathy. Bisoprolol is a beta-1 selective blocker with the highest selectivity for this receptor, in doses less than 10 mg it has very little or no effect on beta-2 receptors. Bisoprolol was first tested in CHF in the CIBIS I trial which enrolled 641 patients and showed improvement in functional class, less hospitalizations for heart failure and a trend to improved survival. The much larger randomized CIBIS II assigned 2647 patients with class III or IV HF and an LVEF <40 percent to bisoprolol or placebo; the patients also received standard therapy with diuretics and ACE inhibitors. After an average follow-up of 1.4 years, the trial was prematurely stopped when the benefits were observed in the active treatment group: significant reduction in total all-cause mortality (11.8 versus 17.3 percent] that was independent of the severity or cause of HF. This benefit was primarily due to a reduction in SCD (3.6 versus 6.3 percent, p < 0.001], with a non-significant trend toward fewer deaths from HF; significant 15 percent reduction in hospital admissions for any cause and a 30 percent reduction in admissions for HF (p < 0.0001). Considering all the beneficial effects beta-blockers in patients with mild to severe CHF due to ischemic and non ischemic disease, there is no data regarding its potential effects in patients with CHF due to Chagas' disease. Based on the aforementioned we propose to develop a multi-center placebo controlled clinical trial to evaluate the effect of *Bisoprolol *in patients with CHF due to CD.

### Consquences of Chagas' disease

CD is the major cause of disability secondary to tropical diseases in young adults from Latin America. In Colombia, 18% of the population lives in CD endemic areas, 900,000 people are infected and over three million are at high risk of being infected. The Northeastern region carries the highest *T. cruzi *infection rate and CD prevalence. However, the incidence of CCM among *T. cruzi *infected people is unknown and the mechanisms that lead from infection to CCM are uncertain. The mechanisms that trigger the development of CCM in 20%–30% of those infected remain a matter of debate.

Despite the high social burden of the disease no large clinical trials have been performed to determine the effects of standard heart failure therapy on hard outcomes in patients with CCM. Experimental and clinical evidence supports the fact that CCM may be primarily an adrenergic cardiomyopathy. Circulating antibodies to beta-adrenergic receptors have recently been described by Esterin-Borda et al providing further support to the "adrenergic hypothesis". A marked reduction in overall mortality and significant improvement in quality of life, HF hospital admissions, reduction in sudden cardiac death have been reported by the mayor clinical trial using beta-adrenergic blockade. The CIBIS trials using Bisoprolol have definitely established the beneficial effects of this compound in patients with both ischemic and non-ischemic dilated cardiomyopathy and HF. We therefore hypothesize that administration of Bisoprolol compared to placebo in patients with CCC on standard HF therapy (Digoxin, ACEi, Diuretics) will have a 30% RR in a specified predetermined composite outcome.

### Expected impact

We expect that Bisoprolol in patients with CCM will determine a 30% relative risk reduction in mortality and morbidity. In the long term, we expect that this project will contribute to reduce the impact of the CCM and the health burden on the Colombian's health care system, particularly related with HF secondary to CCM.

### Direct and indirect user

The direct users will be the seropositive subjects for *T. cruzi*, with different NYHA functional class of heart failure that will recieve b-blocker therapy, which may reduce the morbidity and mortality in these patients. Other direct users will be all physicians in charge of managing these patients. They may have more accurate information regarding the current managment of Chagas' disease. The indirect user will be the Colombian health policy developers which will be benefited by the results of this study, in order to decrease the economic burden that CCM has on the Colombian health care system.

## Methods

### Objectives

In order to achieve these objectives we defined primary and secondary outcomes which apply to this specific cardiomyopathy, addressing the higher incidence of ventricular arrhythmias and heart block.

### Primary objective

To investigate the effect of the beta-blocker bisoprolol on survival and HF hospitalization rates, and other previously predefined outcomes in patients with Chagas' cardiomyopathy and heart failure.

### Secondary objective

To investigate the effect of the beta-blocker bisoprolol on quality of life, conduction disease progression and need for electrical therapy (i.e. pacemaker, ICD, CRT).

### Hypotheses

1. Bisoprolol will reduce cardiovascular death in patients with Chagas' cardiomyopathy with heart failure functional class NYHA II to IV.

2. Bisoprolol is safe in patients without advanced heart block.

3. Bisoprolol will significantly reduce hospitalization rates due to heart failure in patients with Chagas' cardiomyopathy in functional class NYHA II to IV.

### Study design

CHARITY is a multicenter randomized prospective, double blind, placebo-controlled, forced-titration study in subjects with CHF secondary to CCM.

### Study development

The study will last 2 years. Patient recruitment will take 12 months with an average follow-up time of 1.5 years per patient.

1. Months 0 to 2: Training of the Personal and techniques standardization

2. Months 2 to 6: Patients recruitment and data recollection

3. Months 6 to 24: Follow up of patients

4. Months 24 to 30: Data analysis

Those subjects with positive serology for *T. cruzy *who meet the inclusion/exclusion criteria will receive placebo or Bisoprolol in addition to standard therapy for chronic heart failure which includes an ACE-inhibitor and may include diuretics or other medication such as digitalis or nitrates for heart failure. Patients included in CHARITY should be on stable doses of an ACE-inhibitor. Diuretics can be added to treatment as needed but patients must be on stable doses of each drug during four weeks prior to randomization.

500 subjects will be randomized in two arms; one will receive oral bisoprolol and the other placebo, during the duration of the study. Forced titration will start with the lowest oral dose of 2.5 mg qd and will be up-titrated in 2.5 mg increments every two weeks until the maximum tolerated dose is achieved or 10 mg qd is reached. In case of intolerance, dose will be reduced to the previously tolerated lower dose. Subjects not tolerating the lower dose of 2.5 qd will have a treatment interruption. The best tolerated dose will be continued for one month, then a security visit will re-asses tolerance. Thereafter patients will be controlled every three months until they complete the mean follow-up period of two years. Standard heart failure therapy with ACE-inhibitors, diurectics, digitalis and nitrates will be strongly reinforced in both parallel arms.

### Blinding

Patients and investigators will remain blinded during the trial. The treatment codes will remain blinded until the database is locked for final analysis.

### Pretreatment considerations

Baseline vital signs will be obtained with patients in the sitting and after 3 minutes in the standing position.

Twelve-lead ECGs will be recorded in each patient.

Left ventricular ejection fraction at rest will be determined by echocardiography, using a modified Simpson's rule to calculate LV volumes.

Quality of life questionnaire will be administrated two weeks apart during baseline examination using a translated-validated version of the "Minnesota living with heart failure" questionnaire.

Minimum of two 6-minutes corridor walk test once a week over a 2-week period will be performed. The last value will be used if it is within 10% of the previous value.

### Follow-up period

ECG tracings will be recorded during the security visit and at 1 and 2 years.

Patients will be followed for two years after randomization.

Repeat echocardiograms will be recorded at 1 and 2 years of follow-up along with HF Quality of-life questionnaire and six-minute walk tests.

Clinical measurements will be obtained every three months along with an assessment of treatment adherence and tolerance. Mortality and hospitalization events will be ascertained by telephonic interview and verified with hospital charts review, up to two years after randomization.

### Selection and withdrawal of subjects

#### Inclusion criteria

1. Males or females aged 18 to 70 years.

2. Heart failure symptoms NYHA functional class II to IV

3. Left ventricular ejection fraction <40% determined by bi-dimensional echocardiography using modified Simpson's rule for ventricular volumes.

4. Subjects must be on standard and stable outpatient doses of ACEIs or angiotensin II receptor antagonist for at least four weeks.

5. Subjects receiving diuretics must be on a stable dose for at least two weeks.

6. Clinical Euvolemia: as evidenced by absence of rales, no pleural effusion or ascitis and no more than minimal peripheral edema.

#### Exclusion criteria

1. CHF due to ischemic heart disease, valve disease or any other etiology different than CD.

2. Severe aortic insufficiency

3. Baseline advanced AV block defined as Mobitz type 2 or third degree AV block

4. Serum creatinine >2.5 mg/dl.

5. Resting Heart rate less < 45 bpm

6. Known malignancy and other severe disease which shorten life expectancy < 6 months.

7. Subjects with contraindications for beta-blockers: severe obstructive chronic pulmonary disease, asthma, severe pulmonary hypertension, type 1 diabetes mellitus or history of hypoglicemia.

8. Suspected or confirmed chronic infectious disease including HIV and hepatitis B.

9. History of active substance or alcohol abuse within the last year.

10. Clinically significant psychiatric illness which can negatively affect the subject compliance and participation in the trial.

11. Pregnancy or lactation.

12. Organic disease or gastrointestinal surgery which can affect the oral absorption and pharmacodynamics of the medication under study.

13. Enrollment and participation in other active treatment trial within the previous month.

14. Failure to provide written informed consent.

### Interruption of treatment

Trial treatment should be interrupted under the following conditions.

1. The patient decides it is in his/her best interest.

2. The investigator considers it is advisable or in the patient' s best interest.

3. Intolerable adverse experience(s).

4. Presence of life-threatening conditions despite adjustment of therapy.

If study treatment is interrupted, every effort should be made to reinstate treatment as soon as possible and medically acceptable. The re-initiation dose is determined at investigator' s discretion and the dose may be reduced or re-started at the last dose level. Patients with treatment interruption should continue on the protocol's visit schedule and study procedures except drug dispensing.

### Treatments

The FCV Research Institute (Instituto de Investigaciones FCV) will supply ranurated 5 mg bisoprolol tablets and matched placebo needed for the whole duration of the trial. Study drug and placebo have been kindly provided by Merck Colombia.

### Efficacy assessment

Efficacy assessments will be made at every visit during the trial following randomization. Information will be recorded in the supplied CRF formats dealing with clinical variables, Adverse Events (AE), Serious Adverse Events (SAE) and endpoints.

### Primary outcomes

Primary outcomes are the first occurrence of any of the following:

1. Cardiovascular death.

2. Hospital admission caused by heart failure.

3. Major adverse cardiovascular events: stroke, systemic embolism, resuscitated sudden death.

4. Bradycardia requiring pacemaker implantation.

5. Clinically significant sustained monomorphic ventricular tachycardia causing syncope: sustained ventricular tachycardia or ventricular fibrillation.

Each of the primary endpoints will be recorded on the SAE form and in the individual corresponding form for each endpoint.

### Secondary efficacy outcomes

Secondary outcomes are the occurrence of any of the following:

1. Non-cardiovascular death.

2. Heart failure worsening or mortality related with CHF.

3. New AV block.

4. Need for Implantable cardioverter-defibrillator (ICD), Cardiac resynchronization Therapy (CRT) or Pacemaker therapy (PM).

5. Perceived quality of life worsening.

### Endpoint definitions

1. Cardiovascular death: any death attributable to a cardiac or vascular cause occurring within two years after randomization.

2. Non-cardiovascular death: death from any cause excluding cardiovascular diseases occurring within two years after randomization.

3. Hospital admission/hospitalization caused by heart failure: admission in a hospital or clinic during at least 24 hours caused by heart failure worsening. It excludes procedures and hospitalization for procedures which have been scheduled and not due to worsening of the current heart failure condition since randomization.

4. Heart failure worsening: decline of at least one functional class as defined by the New York Heart Association occurring during any inter- visit period of three months needing inotropic support, IV diuretics, medication dose adjustments or additional drugs for compensation.

5. Perceived quality of life worsening: increase in the "Minnesota living with heart failure" test score of at least 10 points. The test' s score ranges from 0 (best) to 100 (worst).

6. New AV block: appearance of any AV conduction disturbance including first, second and third degree AV block after randomization.

7. Bradycardia requiring pacemaker implantation: ventricular rates < 50 per minute due to any cause including sick-sinus syndrome and AV block, causing symptoms or disability and complying with current indications for permanent pacemaker implantation.

8. ICD: Implantable cardioverter-defibrillator indicated for the prevention and treatment of sudden death, ventricular tachycardia/ventricular fibrillation.

9. CRT: cardiac resynchronization therapy or biventricular pacing or ventricular bifocal pacing for the treatment of advanced heart failure.

10. PM: permanent pacemaker for the treatment of conduction disturbances.

11. Clinically significant ventricular arrhythmias: sustained monomorphic ventricular tachycardia or ventricular fibrillation with symptoms caused by hemodynamic or perfusion alterations, circulatory collapse or syncope.

### Monitoring committees

#### Steering committee

The Steering Committee is composed of a group of national leaders who will mostly be cardiologists, since patients with CHF and Chagas' disease are most often cared for by cardiologists. This group will be supplemented with experts in neurology, electrophysiology, coagulation and thrombosis.

The committee has the overall responsibility for producing and conducting a scientifically sound design and ensuring accurate reporting of the study. In that capacity, the Steering Committee must address and resolve scientific issues encountered during the study. This committee will meet at least twice a year. The main role of the Steering Committee is the development of the protocol and CRF and to ensure appropriate conduction of the trial. The steering committee is composed by the principal investigators and site investigators. The chairman of the steering committee is the principal investigator. All proposed ancillary research investigations on patients enrolled in CHARITY must be approved by the Steering Committee. The primary scientific publication reporting the study results is the responsibility of the Steering Committee. Collaborating Investigators or members of the various study committees wishing to prepare secondary publications must submit proposals and manuscripts to the Steering Committee for approval. The final decision on the contents of all publications will be the responsibility of the CHARITY Operations Committee.

#### CHARITY Operations Committee

The Operations Committee will consist of a select group of Steering Committee members chosen for their specific expertise and experience. This group will be responsible for ensuring that study execution and management are of the highest quality. The Operations Committee will convene regularly by teleconference and/or face-to-face meeting (at least every 2 months) to discuss and report on the ongoing supervision of the study. Issues relating to regulatory reporting are the responsibility of the Sponsor, although the Operations Committee is to be kept informed of these activities. The Operations Committee will determine its own guidelines and approve the criteria and guidelines of the other committees prior to commencement of the study. The Operations Committee will need to determine its own working guidelines, especially regarding the decision-making process on specific aspects of the study conduct. Throughout the trial, the Operations Committee will monitor the overall number of confirmed primary events and may decide to increase the sample size in order to maintain or enhance the statistical power of the study.

### FCVRI project office

The FCVRI Project Office is located at the Instituto de Investigaciones/FCV in Bucaramanga, Santander, Colombia. The FCVRI Project Office is independent and its primary function is to facilitate and oversee the execution of the study. The FCV Project Office will keep the Operations Committee appraised of the progress and conduct of the trial and will provide ongoing administrative and methodological support to the Event Adjudication Committee and the DSMB. It will also make available appropriate study data and/or documentation to these committees.

Oversight activities of the FCCVProject Office include the following :

1. Develop and manage the Central Study Database, perform data entry and validate records in the DataFax system:

2. Implement and manage the central randomization system;

3. Generate patient visit schedules for all centres;

4. Compile and generate monthly study status reports for general distribution to all personnel involved in the study, centrally and locally;

5. Maintain necessary communication with all National Investigators and study personnel in order to provide methodological and administrative assistance to them, so that the requirements of a well-disciplined and successful trial can be fulfilled;

6. Provide appropriate reports every two weeks in order to facilitate and optimize site monitoring and study drug distribution;

7. Perform the final analysis for scientific publication.

The FCVRI Project Office will also collaborate in the following areas:

1. Ensure protocol adherence;

2. Assess timely completion and submission of Case Report Forms (CRFs) and support documents;

3. Monitor quality and homogeneity of all data processing activities;

4. implement appropriate measures for data quality control.

As part of the overall study management, the FCVRI Project Office will prepare and distribute monthly status reports to all collaborators at the central and local level, including investigators. The primary purpose of these reports will be to keep all Investigators informed of the performance at their Clinical Centre in relation to the combined performance of the other participating Clinical Centres. These reports will contain the following information:

1. Patient accrual rates

2. Permanent study drug discontinuations

3. Overdue clinical assessments

4. Projected patient follow-up schedules

### Data and Safety Monitoring Board (DSMB)

CHARITY will be conducted in a double-blind manner in which patients and treating physicians are blinded. The trial management team (Operations Committee, Event Adjudication Committee, and the FCVRI Project Office) will also be blinded with respect to treatment allocation. The DSMB will include at least 2 prominent cardiologists and a neurologist, as well as a statistician. Their mandate will be to provide on-going review of the safety of all the investigational treatments. To facilitate its responsibilities, the DSMB will have an Associated Statistician who will receive study data directly from the Central Study Database and who will remain independent of the trial management team. The DSMB Associated Statistician is not a member of the DSMB, but presents data to the committee and is responsible to the Chairman. The DSMB Associated Statistician, being unblinded, will not be able to edit/alter any part of the Central Study Database. Routine access to the treatment code will be restricted to the Chairman of the DSMB, except for emergency unblinding on a case by case basis.

#### DSMB responsibilities

##### Primary

1. Regular (at least every 3 months) review of safety data and serious adverse events

2. Formal interim analyses of efficacy data

3. Feedback to the Operations Committee

##### Secondary

1. Respond to special requests from regulatory authorities or IRBs

2. Recommendations for protocol amendments

3. Verification of final analysis of the study will be done by the DSMB Associated Statistician

Recommendation to stop a trial early for safety reasons is, by definition, a qualitative judgment. The DSMB is composed of eminent clinicians and methodologists who are experienced with clinical trials and can be relied upon to exercise good judgment in weighing the potential risks and benefits to patients as data accumulate in this trial. Safety aspects and more specifically severe bradycardia, heart block, severe hypotension will be monitored. No formal boundaries will be proposed, but clear, consistent, and persistent evidence of net harm that overwhelms any benefit should be apparent. A recommendation to stop the trial will be based on the pattern of treatment effect across all endpoints, as well as the benefit/risk ratio.

The DSMB will fulfill its responsibility to monitor the safety of patients in CHARITY by conducting formal reviews of accumulated safety and efficacy data. These reviews will normally occur at regular intervals. The DSMB Associated Statistician will prepare a report of aggregate data summaries and individual patient data listings, where appropriate, for each treatment group. This report will be circulated to each member of the DSMB at least one week prior to their collective review. The committee will then convene, either by face-to-face meeting or by telephone conference call, to make its recommendation to the Operations Committee with respect to continuance of the trial. A formal written communication to the Chairman of the Operations Committee will then follow. Minutes of all official meetings of the DSMB will ultimately be part of the Sponsor's master files (archives). The report of data summaries and listings will include information on both safety and efficacy parameters, together with status reports designed to show the extent to which the trial is being executed according to protocol. Included among the safety data will be (a) all deaths and (b) adverse events. Efficacy summaries will provide information on the occurrence of study outcomes. The outcome events are considered as distinct from adverse events and summarized separately. At each review by the DSMB, consideration of a decision to stop the trial on grounds of patient safety will weigh the current evidence of differences between treatments regarding adverse effects (as expressed by mortality, adverse event reports, etc.) against emerging trends in efficacy. Providing efficacy data at each of these routine safety reviews does not constitute a formal interim analysis of efficacy. Blinded data for the safety review will be made available from the Central Study Database. Serious adverse events are required to be reported rapidly to the FCVRI Project Office and will be entered into a separate data base. These reports will be added to the data to be reviewed by the DSMB. Outcome events will be reviewed by the Event Adjudication Committee on an ongoing basis to determine if each reported event meets the defined criteria for a study outcome event. The DSMB will review data on all reported outcome events. Additional summaries will show the results of the Event Adjudication Committee's judgements on the subset of reports that has been reviewed by this committee. This committee is independent from the FCVRI and its responsibilities are to review the protocol and perform an interim analysis plan, review safety, efficacy, compliance and trial progress at 12 months after the first randomization and if requested by the Steering Committee. The Data and Safety Monitoring Board (DSMB) will make recommendations to the Steering Committee about any potential problems. Any major recommendation, e.g. amending the protocol or stopping the trial will be reviewed and ratified by the Steering Committee.

### Adverse Events and Adjudication Committee

The Event Adjudication Committee is charged with the responsibility for validating all reported primary fatal and nonfatal outcomes and validating the classification of cause of death. The Event Adjudication process will be coordinated at the FCVRI Project Office in Bucaramanga. This committee, composed of experts in the field will review, in a blinded manner; all reported outcome events to provide consistency and validity in the assessment of outcomes. Their decisions will be based on blind clinical data provided and they will consider the impressions of the clinical investigator. Their decisions will be used in the final analysis. Members of the Event Adjudication Committee will be chosen based on their clinical expertise. Adjudicators will be trained at a preliminary meeting where study definitions will be reviewed and test cases performed to ensure uniform application of study definitions. Reported events will be adjudicated by at least one committee member. Dossiers of reported events will be prepared and distributed to committee members on a regular basis to ensure that events are adjudicated in a timely fashion. Each committee member will be requested to review the dossiers and acknowledge in writing their agreement/disagreement with the investigator's interpretation of events.

The AE Committee (AE&AC) is composed by clinical experts and one principal investigator, its responsibilities are to review and verify every AE, SAE and Adverse Drug Reactions (ADR) reported, and to supervise all relevant SAE information is complete. The AE&EC will validate all SAE in a blinded form and notify investigators, Independent Ethics Committees and regulation authorities of all SAE and clinically relevant AE. The AE&EC will also validate outcomes providing standard classifications and definitions and reviewing supporting information provided by investigators.

### Safety assessment

Safety assessment will consist of monitorization of all adverse events, serious adverse events and protocol outcomes. Physical exam including sitting and standing blood pressures, heart rate and presence of CHF signs will be measured and recorded in the case report form (CRF). During visit 4, the security visit, an ECG will be recorded to assess heart rate and presence of conduction disturbances before proceeding to dispense medication.

### Adverse events

All adverse events informed by the subjects, detected by the investigators or detected through physical examination will be recorded in the individual' s CRF and reported to the principal investigators within the next five days.

An adverse Event (AE) is any untoward or undesirable medical occurrence in a subject that does not necessarily have a causal relationship with the study treatment medication. An AE can be any symptom, sign or medical condition occurring after starting the study drug whether it corresponds to bisoprolol or placebo. Medical conditions present before starting the study are considered AE only if they worsen after starting the study treatment. An abnormal laboratory finding not present before starting the study treatment is also considered an AE and should be recorded in the CRF.

The Adverse Events is described in the CRF Adverse Events form (CRF 50) including date and duration, severity (mild, moderate, severe, only laboratory abnormality), relation with the study drug (suspected, not suspected), action taken (none, drug discontinued, dose adjustment, drug interruption, hospitalization, withdrawal) and outcome of the event (recovery, persistent problem, sequelae, death). All fields requested in the AE form must be filled and submitted to the AE committee.

### Effects of Bisoprolol

The known effects and tolerability to bisoprolol will be monitored in each visit; any intolerability or negative change in vital signs will be recorded as an AE. The following definitions apply to the side effects associated with Bisoprolol independent of the study' s endpoints.

1. Bradycardia: Bradycardia is defined as a frequency of <45 beats per minute detected by pulse palpation, cardiac auscultation or ventricular frequency in an ECG.

2. Hypotension: Hypotension is defined as a sitting systolic blood pressure < 100 mmHg or a sitting diastolic blood pressure < 60 mmHg. The investigator must sate whether hypotension is asymptomatic or symptomatic.

3. Orthostatic hypotension: Orthostatic hypotension is a decrease in systolic or diastolic blood pressure of ≥20 mmHg or ≥10 mmHg respectively. The investigator must sate whether orthostatic hypotension is asymptomatic or symptomatic.

4. Fatigue: Fatigue is described as a perceived sensation of weakness during ordinary physical activities not present before starting the study treatment drug.

5. Bronchospasm: Bronchospasm is defined as bronchial wheezing during auscultation or wheezing perceived by the subject during respiratory symptoms of cough or dyspnea.

6. Cold extremities: Cold extremities are defined as the sensation of coldness in digits, hands or feet not present before starting the study drug.

### Serious Adverse Events

Serious Adverse Event (SAE) is any untoward medical occurrence that at any dose of the study drug results in:

1. Death

2. Is life-threatening

3. Requires hospitalization or prolongation of existing hospitalization

4. Results in persistent or significant disability

5. Results in congenital anomaly at birth

SAE will be recorded in the CRF SAE form and reported to the principal investigator at FCV within 24 hours of learning of its occurrence. The SAE form requires information about date, medical diagnosis, consequences and severity (fatal, life-threatening, requiring hospitalization, disability/incapacity, birth defects or other consequence), date of hospitalization, date of death, study treatment drug dose, date of first dose, date of dose adjustment, relationship with the study treatment drug (suspected, not suspected) and study withdrawal of the subject (yes or no). The SAE form must be signed by the principal investigator and the study monitor.

All SAE will be followed until their resolution or the completion of the trial. Follow-up will be recorded in the CRF SAE follow-up (CRF52) form stating the subject' s outcome as resolved, dead, lost to follow-up or unknown. Hospitalization SAE should include copy of the summary epicrisis from the corresponding hospital or clinic. All SAE follow-up forms must be signed by the principal investigator and the study monitor.

SAE which constitute endpoint according to the protocol' s definitions, will be recorded additionally in the CRF corresponding endpoint form. Each endpoint has its own CRF page which must be submitted to the AE committee for validation.

Events not considered SAE are hospitalization for:

1. Routine treatment or pre-scheduled hospitalizations for heart failure not associated with any clinical deterioration.

2. Elective or scheduled treatment for pre-existing conditions that did not worsen during the study.

### Statistical methods

Descriptive statistical analysis will be composed with simple distribution of frequencies, calculation of proportions, means, their respective standard deviations and 95% confidence intervals. For effects of group comparison, t-test and Mann-Whitney tests will be used according to the dependent variable distribution. For categoric variables, the Chi-square test or the exact Fisher test will be applied as corresponding. Patient survival and hospitalization rates will be described using Kaplan-Meijer estimates and survival graphs. Cox regression will be used for the multivariate analysis of time to death and time to hospitalization. Formal efficacy interim analyses will take place one year after the recruitment phase.

### Ethical aspects

This study will be conducted in accordance with the Declaration of Helsinki and with the Colombian legislation as per the Resolution 8430/93 from the Ministry of Health. Prior to the admission of the patients in the study, the objectives and the methodology will be explained and the informed consent obtained. The study was approved by the Research Ethic Committee of the Cardiovascular Foundation of Colombia (No. 059/June 29/2003). The right to confidentiality of the patients will be maintained in all the phases of the study.

## Competing interests

The author(s) declare that they have no competing interests.

## Authors' contributions

CAM, LAC, and JPC, conceived and designed the study, and will be responsible for the overall administration and direction of the project. FRQ has made significant contributions in the recruitment and follow-up of patients. The analysis and interpretation of data and will be performed by all authors and give the final approval of the version to be published. FAS, contributed to the conception and design of the study, performed sample size calculations and randomization and will analyze the data obtained from the clinical trial. All authors read and approved the final manuscript.

## References

[B1] Araujo RD, Bestetti Rb, Godoy RA (1985). Chronic Chagas' heart disease in children and adolescents: a clinicopathologic study. In J Cardiol.

[B2] Pinto Dias JC (1988). Epidemiology of Chagas' disease in Brazil. In: Brenner RR, Stoka AM, eds. Chagas' disease vectors.

[B3] Coura JR (1976). Evolutive patterns in Chagas' disease and the life span of Trypanosome cruzi in human infections. In: New approaches in American trypanosomiasis research.

[B4] (1991). UNDP/World Bank/WHO Special Programme for Research and Training in Tropical Diseases, Tenth Programme Report,. Chagas' disease.

[B5] WHO (1985). Demographic YearBook 1983.

[B6] PanAmerican Organization (1984). Status of Chagas' disease in the region of the Americas. Epidemiologic bulletin.

[B7] Hagar JM, Rahimtoola MB (1995). Current Problems in Cardiology. Chagas' Heart Disease.

[B8] Roberti RR, Martinez EE, Andrade JL (1992). Chagas' Cardiomyopathy and Captopril. Eur Heart J.

[B9] Morris SA (1984). Alteration of the pattern of beta-adrenergic desensitization in cultured L6E9 muscle cell infected with Trypanosome cruzi. Mol Biochem Parasitol.

[B10] Willette RN, Aiyar N, Yue TL (1999). In vitro and in vivo characterization of intrinsic sympathomimetic activity in normal and heart failure rats. J Pharmacol Exp Ther.

[B11] Levy P, Lechat P, Leizorovicz A (1998). A cost minimization of heart failure therapy with bisoprolol in the French setting: an analysis from CIBIS trial data. Cardiac Insufficiency Bisoprolol Study. Cardiovasc Drugs Ther.

[B12] CIBIS II Investigators and Committees (1999). The Cardiac Insufficiency Bisoprolol Study II: a randomized trial. Lancet.

[B13] CIBIS Investigators and Committees (1994). A randomized trial of b-blockade in heart failure. The Cardiac Insufficiency Bisoprolol Study. Circulation.

[B14] Kukin ML, Kalman J, Charney RH (1999). Prospective, randomized comparison of effect of long term treatment with Metoprolol or Carvedilol on symptoms, exercise, ejection fraction and oxidative stress in heart failure. Circulation.

[B15] Australia-New Zealand Heart Failure Research Collaborative Group (1995). Effects of Carvedilol in patients with congestive heart failure due to ischemic heart disease. Circulation.

[B16] Packer M, Bristow MR, Cohn JN (1996). The effect of Carvedilol on morbidity and mortality in patients with chronic heart failure. N Engl J Med.

[B17] Bristol Mr, Giblet EM, Abraham WT (1996). Carvedilol produces dose related improvements in left ventricular function and survival in subjects with chronic heart failure. Circulation.

[B18] Colucci WS, Packer M, Bristow MR (1996). US Carvedilol Heart Failure Study. Circulation.

[B19] Packer M, Colucci WS, Sackner-Bernstein JD (1996). Prospective Randomized Evaluation of Carvedilol on Symptoms and Exercise. Circulation.

[B20] Haggar J, Rahimtoola S (1991). Chagas' Heart Disease. N Engl J of Med.

[B21] Waagstein F, Hjalmarson A, Varnauskas E (1975). Effect of chronic beta-adrenergic receptor blockade in congestive cardiomyopathy. Br Heart J.

[B22] Mady C, Antonelli RH, Pereira AC Survival and Predictors of survival in patients with congestive heart failure due to Chagas' Cardiomyopathy. Circulation.

[B23] Packer M, Coats A, Fowler M (2001). Effect of Carvedilol on survival in severe chronic heart failure. N Engl J of Med.

[B24] Dargie HJ (2001). Effects of Carvedilol on outcome after myocardial infarction in patients with left-ventricular dysfunction: The Carpicorn randomized trial. Lancet.

[B25] Packer M, Cohn J (1999). Consensus recommendations for the management of chronic heart failure. Am J of Cardiol.

[B26] Remme W, Swedberg K (2001). Guidelines for the diagnosis ams treatment of chronic heart failure. Eur Heart J.

